# Bone Marrow Osteoblast Damage by Chemotherapeutic Agents

**DOI:** 10.1371/journal.pone.0030758

**Published:** 2012-02-17

**Authors:** Stephanie L. Rellick, Heather O'Leary, Debbie Piktel, Cheryl Walton, James E. Fortney, Stephen M. Akers, Karen H. Martin, James Denvir, Goran Boskovic, Donald A. Primerano, Jeffrey Vos, Nathanael Bailey, Marieta Gencheva, Laura F. Gibson

**Affiliations:** 1 Cancer Cell Biology Program, School of Medicine, West Virginia University, Morgantown, West Virginia, United States of America; 2 Alexander B. Osborn Hematopoietic Malignancy and Transplantation Program, School of Medicine, West Virginia University, Morgantown, West Virginia, United States of America; 3 Department of Microbiology, Immunology and Cell Biology, School of Medicine, West Virginia University, Morgantown, West Virginia, United States of America; 4 Department of Pediatrics, School of Medicine, West Virginia University, Morgantown, West Virginia, United States of America; 5 Department of Neurobiology and Anatomy, School of Medicine, West Virginia University, Morgantown, West Virginia, United States of America; 6 Department of Statistics, School of Medicine, West Virginia University, Morgantown, West Virginia, United States of America; 7 Microarray Core Facility, Marshall University, Huntington, West Virginia, United States of America; 8 West Virginia University Department of Pathology, School of Medicine, West Virginia University, Morgantown, West Virginia, United States of America,; University of Frankfurt - University Hospital Frankfurt, Germany

## Abstract

Hematopoietic reconstitution, following bone marrow or stem cell transplantation, requires a microenvironment niche capable of supporting both immature progenitors and stem cells with the capacity to differentiate and expand. Osteoblasts comprise one important component of this niche. We determined that treatment of human primary osteoblasts (HOB) with melphalan or VP-16 resulted in increased phospho-Smad2, consistent with increased TGF-β1 activity. This increase was coincident with reduced HOB capacity to support immature B lineage cell chemotaxis and adherence. The supportive deficit was not limited to committed progenitor cells, as human embryonic stem cells (hESC) or human CD34+ bone marrow cells co-cultured with HOB pre-exposed to melphalan, VP-16 or rTGF-β1 had profiles distinct from the same populations co-cultured with untreated HOB. Functional support deficits were downstream of changes in HOB gene expression profiles following chemotherapy exposure. Melphalan and VP-16 induced damage of HOB suggests vulnerability of this critical niche to therapeutic agents frequently utilized in pre-transplant regimens and suggests that dose escalated chemotherapy may contribute to post-transplantation hematopoietic deficits by damaging structural components of this supportive niche.

## Introduction

The stem cell niche hypothesis was first presented in 1978 by Schofield who suggested that stem cells were associated with accessory cells that influence their behavior [1]. Studies from several labs have expanded our appreciation of the unique anatomical niches within the marrow microenvironment and have characterized areas of optimal stem cell support [2]. The niche's cellular components consist of osteoblasts (HOB), bone marrow stromal or mesenchymal stem cells (BMSC, MSC), and endothelial cells [3], [4]. Recent work has demonstrated the importance of the interaction of HOB and stem cells in the niche, suggesting that hematopoietic stem cells (HSC) can regulate MSC differentiation into HOB and that they, in turn, play an important role in the support of B lymphocytes and differentiation of HSCs [5], [6]. Additionally, it has been shown that resting HSCs are maintained in a quiescent state as a result of their close proximity to HOB and that the number of HSCs change as a result of the number and type of HOB present [7], [8], highlighting the potential for hematopoietic deficiencies if HOB function is altered. Studies describing BMSC have shown that damage by chemotherapy and radiotherapy can affect the ability of the BMSC to self-repair and leads to decreased numbers of functional immune system cells in the blood, with deficits persisting years after transplant [9], [10]. The effects of chemotherapy on HOB have been studied in some detail by other groups, but not the extent of BMSC. Studies by Davies et al. used both osteoblast-like cell lines and osteoprogenitor cell lines and showed that exposure of these cells to chemotherapeutic agents led to decreased cell numbers, and interestingly, osteoprogenitor cells appeared to be deleted preferentially [11]. Davies et al. also demonstrated that combination chemotherapy commonly used in the treatment of childhood malignancies led to decreased HOB numbers, which could be restored by administering glucocorticoids [12]. Using a rat model, Xian et al. studied the effects of methotrexate on bone growth and they observed that methotrexate exposure led to many different types of bone damage, but this affect could be abrogated with the addition of folinic acid, which promoted proliferation of osteoblast progenitors [13]. Finally, Fan et al. described how different chemotherapeutic agents affect bone growth differently and described the implications that using these agents in combination might have post-therapy [14]. While these studies have focused on the affects of chemotherapy on bone growth and recovery, most have not investigated how this chemotherapy-induced damage to HOB affects HSC and progenitor cell recovery and support following transplantation and further investigation is warranted.

The stem cell niche is characterized, in part, by expression of specific cytokines, including TGF-β and CXCL-12, to facilitate signaling between the niche components and HSC. Studies have demonstrated that chemotherapy increases the levels of active TGF-β1 resulting in decreased ability of BMSC to support HSC [15], [16]. It has also been shown that TGF-β1 has crosstalk with CXCL-12 and can stimulate the differentiation of progenitor cells to erythroid and myeloid cells resulting in a deficit of the primitive stem cell pool [17]. The importance of CXCL-12 is emphasized, in part by its requirement for homing of progenitor cells to the bone marrow following transplantation [18], [19]. We have previously demonstrated that diminished levels of CXCL-12 in the supernatants of VP-16 treated BMSC results in the loss of an optimal chemokine gradient to which CXCR-4+ pro-B cells respond with CXCL-12, subsequently shown to also be important in regulation of stem cell phenotype by Guo et al. [20], [21]. Sugiyama et al. showed that mice deficient in the CXCL-12 receptor, CXCR-4, had a reduction in HSC, in both vascular and endosteal niches, and increased sensitivity to myelotoxic stress compared to their wild-type counterparts [22]. Other studies of CXCR-4 in the HSC niche have shown that CXCR-4 is essential to maintain quiescence and retention of stem cells [23].

In the current study, we investigated melphalan and VP-16 induced damage to HOB with emphasis on CXCL-12 and TGF-β1 levels following exposure to chemotherapeutic agents, and the implications of this damage on hematopoietic reconstitution. Global changes in HOB gene expression in response to melphalan were investigated to determine the vulnerability of HOB to genotoxic stress. In addition, TGF-β1, CXCL-12 and VCAM-1 were investigated as representative HOB proteins involved in three critical functions of the endosteal niche; support of pluripotency, homing and stem cell retention [24], [25]. Our results indicate diverse changes in gene expression profiles following HOB exposure to melphalan, conditioned media from BMSC pre-treated with melphalan, and following exposure to rTGF-β1 as one of the factors elaborated by chemotherapy damaged BMSC [15]. Coincident with altered gene expression profiles, HOB exposed to chemotherapeutic agents or rTGF-β1 had increased levels of active TGF-β1, altered support of differentiation of CD34+ bone marrow cells, reduced ability to support Oct-4 positive embryonic stem cell colonies, and reduced chemotactic support and adhesion of CXCR-4+ pro-B cells. These data suggest that the niche in which hematopoietic recovery occurs following transplantation may be more vulnerable to damage than previously appreciated.

## Materials and Methods

### Cell Lines and Reagents

Human osteoblasts (HOB) were purchased from Promocell (Heidelberg, Germany) and maintained in osteoblast growth media. The CXCR-4+/VLA-4+ pre-pro-B leukemic cell line JM-1 was purchased from the ATCC (CRL-10423, Manassas, VA). The BMSC and IL-7 dependent murine pro-B cell line C1.92 was kindly provided by Dr. Kenneth Landreth and has been described in detail [26]. Human embryonic stem cells (H9, WiCell, Madison WI) were maintained on irradiated mouse embryo fibroblasts (MEF) and grown in DMEM-F12 media (Mediatech, Manassas, VA) supplemented with Knockout Serum Replacement (Gibco/Invitrogen, Carlsbad, CA) 2 mM L-glutamine (Mediatech), 0.05 µM 2-mercaptoethanol (Sigma-Aldrich, St. Louis, MO), non-essential amino acids and B-FGF solution. H9 cells were moved to HOB feeder layers where indicated. CD34+ bone marrow cells (ALLCELLS, Emeryville, CA) were grown in RPMI-1640 supplemented with 10% fetal bovine serum. Melphalan (Sigma-Aldrich) was reconstituted at a stock concentration of 50 mg/ml immediately prior to use. VP-16 (Etoposide, Bristol Myers Squibb, New York, NY) was stored at a concentration of 33.98 mM and diluted immediately prior to use. Human rTGF-β1 (R&D, Minneapolis, MN) was used at a concentration of 10 ng/ml. Human rIL-3 (R&D) was used at a concentration of [100 ng/ml].

### Adhesion assay

HOB were pre-exposed to melphalan [50 µg/ml], VP-16 [50 µM], or rTGF-β1 [10 ng/ml] for 24 hours. C1.92 pro-B cells were stained with CellTracker™ Green CMFDA (Invitrogen) according to the manufacturer's instructions. The HOB adherent layer was thoroughly rinsed following treatment and fluorescently labeled C1.92 pro-B cells (1×10^5^) were added in co-culture for 4 hours. Subsequently, the media containing non-adherent C1.92 was aspirated and the cultures were gently rinsed. Remaining HOB and adherent C1.92 were trypsinized and C1.92 cells were enumerated using a FACSCalibur flow cytometer (BD, Franklin Lakes, NJ) with events counted for 30 seconds on high flow rate. Data were analyzed using WinMDI software.

### Chemotaxis assays

HOB were plated in the bottom chamber of a transwell at 100% confluence and were left either untreated or exposed to melphalan [50 µg/ml], VP-16 [50 µM], or rTGF-β1 [10 ng/ml] for 24 hours. After 24 hours, 350 µL of supernatant was placed in the bottom of a transwell and JM-1 cells (1×10^6^ cells/ml) were added to the top chamber, and incubated at 37°C for 4 hours. JM-1 cells migrated through the 5 µm pores to the bottom chamber towards media supplemented with 100 ng/mL CXCL-12 (positive control), media alone (negative control), or media conditioned by untreated HOB or HOB exposed to melphalan, VP-16 or rTGF-β1 with and without rCXCL-12 [1 ng/ml], which was added prior to addition of CXCR-4+ cells to the top chamber. Cells were collected using a FACSCalibur flow cytometer (BD) with events counted for 30 seconds on high flow rate. Data were analyzed by WinMDI software.

### Colony Assays

To complete colony assays, HOB were left untreated or exposed to melphalan [50 µg/ml] or rTGF-β1 [10 ng/ml] for 24 hours. Following exposure, HOB layers were rinsed and CD34+ bone marrow cells (1.75×10^5^) were added in co-culture supplemented with recombinant IL-3 [100 ng/ml] for 48 hours. CD34+ cells were collected, viability determined, and 1×10^3^ viable cells added to Methocult-Classic (Stem Cell Technologies) (supplemented with rhSCF, rhGM-SCF, rhIL-3 and rhErythropoietin for myeloid lineage) in triplicate. Colonies were counted and scored after 7 days in culture.

### ELISA

To complete the CXCL-12 ELISA (R&D), HOB were plated at 100% confluence and left untreated or exposed to melphalan [50 µg/ml], VP-16 [50 µM] or rTGF-β1 [10 ng/ml] for 24 hours. The media was then removed, cells were rinsed, fresh media added and allowed to condition for 48, 72 or 96 hours post-treatment. Supernatants were collected and a CXCL-12 ELISA completed. The TGF-β1 ELISA (R&D) was completed using HOB plated at 100% confluence in serum free media and left untreated or exposed to rTGF-β1 [10 ng/ml] for 24 hours. The media was removed, cells were rinsed, and fresh serum-free media was added to each well. After 24 hours, supernatants were collected and a TGF-β1 ELISA completed.

### Fluorescent Microscopy

HOB were cultured on coverslips and left untreated or exposed to melphalan [50 µg/ml], VP-16 [50 µM] or rTGF-β1 [10 ng/ml] for 24 hours, washed thoroughly and H9 stem cells added. Co-cultures were monitored daily and upon colony formation, counts based on colony morphology were completed. Coverslips were subsequently stained for Oct-4. To complete intracellular staining, cells were fixed in 4% paraformaldehyde and permeabilized with 0.5% Triton-X-100. After blocking for 30 minutes in 5% BSA/1X PBS, cells were incubated with mouse α-human primary antibody (1 µg), specific for human Oct-4 (Millipore) or matched isotype control, in 5% BSA/1X PBS for 1 hour at RT. Coverslips were washed with 1X PBS, incubated with Alexa Fluor™ 488 labeled secondary α-mouse antibody (1 µg) at RT for 1 hour and mounted on glass microscope slides with ProLong™ Gold plus DAPI (Invitrogen). Confocal images were acquired using a Zeiss LSM510 confocal system connected to a Zeiss AxioImager microscope (Thornwood, NY). Photographs of human embryonic stem cells were taken using a Nikon Coolpix 990 camera. To complete phospho-Smad2 staining, HOB cells were plated on coverslips and left untreated or exposed to melphalan [100 µg/ml] or VP-16 [100 µM] for 4 hours. Staining and imaging was completed as described above using a murine primary antibody (1.5 µg/coverslip), specific for human phospho-Smad2 (Cell Signaling Technology Inc., Danvers, MA) or matched isotype control.

### HOB and CD34+ cell co-culture

HOB were grown to confluence and were left untreated or exposed to melphalan [25 µg/ml] or rTGF-β1 [10 ng/ml] for 24 hrs. Following treatment, HOB were rinsed and human CD34+ bone marrow cells were added. Cultures were supplemented with recombinant IL-3 (100 ng/ml) every 2 days. CD34+ bone marrow cells were collected after 48 hours in co-culture. Viability was determined and staining for the following cell surface makers was completed using the indicated antibodies: phycoerythrin (PE)–conjugated CD4 (clone SK3), CD19 (4G7), phycoerythrin-Cy7 (PE-Cy7)–conjugated CD56 (NCAM16.2), CD34 (8G12), peridinin-chlorophyll protein-Cy5.5 (PerCP-Cy5.5)–conjugated CD3 (SK7), CD33 (P67.6), fluorescein isothiocyanate (FITC)–conjugated CD8 (SK1), CD71 (L01.1), allophycocyanin (APC)–conjugated CD14 (MϕP9), CD3 (SK7), and allophycocyanin-Cy7 (APC-Cy7)–conjugated CD45 (2D1) ([BD], San Jose, CA). The samples were stained in the following combinations: isotype controls and CD45, CD45, CD34, CD3, CD19, CD33, and CD71 and CD45, CD3, CD4, CD8, CD56, and CD14. Data were acquired with a FACSCanto II (BD) flow cytometer with a minimum of 30,000 events for each sample and analyzed with FACSDiva software (BD). Initial gating was based on forward (FSC)/side scatter (SSC) to exclude debris and non-viable events. Thresholds for positivity were set such that greater than 99% of events within the gated region were negative for each isotype-matched control antibody. Positive events were back-gated to ensure that they constituted a discrete population by CD45/SSC, confirming specificity of antigen binding.

### Microarray

HOB were left untreated or exposed to melphalan [50 µg/ml], rTGF-β1 [10 ng/ml] or conditioned media from BMSC (exposed to melphalan [50 µg/ml] for 24 hours) for 6 hours. Total RNA was isolated from HOB using the RNeasy RNA isolation kit (Qiagen,Valencia, CA) with quality assessed by electrophoretic analysis on an Agilent Model 2100 Bioanalyzer. RNA samples had integrity numbers greater than 8.0 (8.4–10). RNA (250 ng) was used as the template for synthesis of internally labeled cRNAs using the Agilent QuickAmp Labeling kit and cyanine 3-CTP and cyanine 5-CTP (Perkin Elmer, Waltham, MA) and a modified QuickAmp protocol [27]. A total of 825 ng of cyanine 3- and cyanine5-labeled cRNAs was combined and hybridized onto Agilent Whole Human Genome 4×44 K microarrays at 65°C for 17 hours and washed according to the manufacturer's protocol. Slides were scanned on an Agilent DNA Microarray Scanner. HOB exposed to rTGF-β1 and conditioned media were competitively hybridized against untreated HOB in a balanced block design with six replicates. HOB exposed to melphalan and untreated HOB were hybridized against Stratagene Universal Reference RNA (Agilent Technologies, Santa Clara, CA) in a universal reference design with four replicates.

Intersections of groups and corresponding statistically significant fold changes (details described in the supplemental section) for each experiment were imported into Ingenuity Pathway Analysis (IPA) software v 2.6 (Ingenuity Systems ®, Redwood City, CA, www.ingenuity.com). A core analysis was performed in IPA, using default settings, to search for networks associated with these lists of genes. MIAME compliant complete microarray data may be accessed at the NCBI Gene Expression Omnibus (GEO) database (GSE17860).

### Real Time Reverse Transcriptase PCR

Total cellular RNA was isolated from HOB using the RNeasy RNA isolation kit (Qiagen). Real time RT-PCR was performed using 50 ng RNA per reaction using the QuantiTech SYBR Green RT-PCR kit (Qiagen). Primers specific for human CXCL-12 were obtained from SuperArray (Frederick, MD) with 0.25 µl used per reaction. Primers specific for TGF-β1 and the housekeeping gene (HPRT-1) were purchased from Real Time Primers, LLC (Elkins Park, PA). Samples were analyzed in triplicate using the Applied Biosystems 7500 Real-time PCR system (Foster City, CA). Amplification parameters included 50°C for 30 minutes, 95°C for 15 minutes, 94°C for 15 seconds (×45 cycles), 58°C for 30 seconds, and 72°C for 45 seconds. Changes in gene expression were determined using the Comparative Ct method and analysis of relative gene expression data using real-time quantitative PCR and the 2(-Delta Delta C(T)) Method [28].

### Western Blot Analysis

HOB were grown to confluence and left untreated or exposed to melphalan [100 µg/ml], VP-16 [100 µM] or rTGF-β1 [10 ng/ml] with or without SB 431542 [10 µM] for 4 hours. Following treatment, cells were collected and protein isolated and quantitated. Western blot analysis was completed and probed using antibodies specific for P-Smad2 (Cell Signaling), total Smad-2 (Cell Signaling) and anti-rabbit-HRP (Santa Cruz Biotechnology, Santa Cruz, CA). Proteins were visualized using Immobilon Western ECL reagents (Millipore). Quantitation was completed using Image J software.

### Statistics

Data were analyzed using the Students–t test or ANOVA where appropriate with statistical significance of p≤.05 denoted by an asterisk (_*_). Microarray data analysis is described in the supplemental section.

## Results

### Melphalan and VP-16 exposure of HOB increases the levels of active TGF-β1

Earlier observations suggested that BMSC exposed to chemotherapy have higher levels of active TGF-β1 and diminished capacity to support pro-B cells and normal hematopoiesis [15]–[17], [29]. Additionally, retrospective studies of patients that received allogeneic bone marrow transplants showed that they have serious and irreversible stromal damage as measured by CFU-F frequencies that did not recover to the levels of normal control patients even after 12 years, suggesting that the damage of supportive cells of the bone marrow is sustained [30].

**Figure 1:**
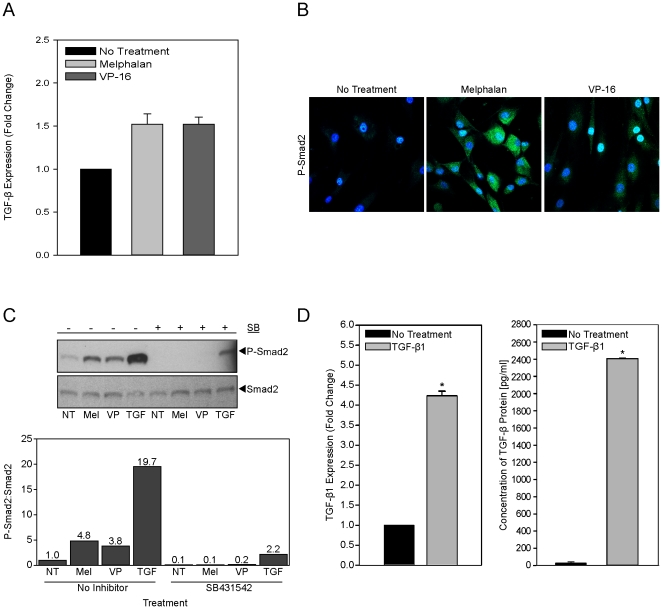
Melphalan and VP-16 exposure of HOB increases the level of active TGF-β1. **A**) HOB were exposed to melphalan [50 µg/ml] or VP-16 [50 µM] for 24 hours and real time PCR for TGF-β1 completed. **B**) HOB were exposed to melphalan [100 µg/ml] or VP-16 [100 µM] for 4 hours, fixed and stained for detection of phospho-Smad2 (green), as a read-out of TGF-β1 activity (DAPI stain for nuclei-blue). **C**) HOB were exposed to melphalan [100 µg/ml], VP-16 [100 µM] or rTGF-β1 [10 ng/ml] with or without SB 431542 [10 µM] for 4 hours and a western blot completed for p-Smad2 and total Smad2. P-Smad2 expression was normalized to total Smad2 expression. **D**) HOB were exposed to rTGF-β1 [10 ng/ml] for 24 hours and real time PCR completed (left). Additionally, HOB were exposed to rTGF-β1 [10 ng/ml] for 24 hours, the HOB layer was rinsed and new media was added and allowed to condition for 24 hours before being evaluated by ELISA to quantitate the amount of secreted TGF-β1 (right). (NT = No Treatment, Mel = melphalan, VP = VP-16, TGF = rTGF-β1, SB = SB 431542).

To determine if direct exposure of HOB to chemotherapeutic agents resulted in increased levels of active TGF-β1, HOB were exposed to melphalan or VP-16 and the expression of total and active TGF-β1 was assessed. Direct exposure of HOB to chemotherapeutic agents (Figure 1A) does not alter the expression of TGF-β1 mRNA. In contrast, exposure of HOB to melphalan or VP-16 (Figure 1B) and melphalan, VP-16 or rTGF-β1 (Figure 1C) resulted in increased active TGF-β1 reflected by increased phosphorylation of Smad2 protein visualized by confocal microscopy (Figure 1B) and western blot analysis (Figure 1C). To determine if the increase in TGF-β1 signaling and Smad2 phosphorylation was due to increased TGF-β1 induced by the chemotherapeutic agents, SB 431542, a selective inhibitor of TGF-β1 receptor kinases was used in combination with melphalan, VP-16 or rTGF-β1 exposure. SB 431542 was able to inhibit both the basal phosphorylation of Smad2 as well as the increase in Smad2 phosphorylation induced by melphalan or VP-16 exposure. Direct exposure of HOB to rTGF-β1 (Figure 1D) increased both TGF-β1 mRNA and protein expression. To mimic the indirect effects of soluble cues elaborated by damaged stroma on HOB, BMSC were exposed to melphalan or VP-16, rinsed, and allowed to condition media that was then placed on HOB that had not been exposed to chemotherapeutic agents. HOB exposed to conditioned media from damaged BMSC have increased levels of phosphorylated Smad2 when compared to their counterparts exposed to conditioned media from untreated stroma (data not shown).

### Pre-exposure of HOB to chemotherapeutic agents or rTGF-β1 alters the ability of HOB to support hematopoietic progenitors

**Figure 2:**
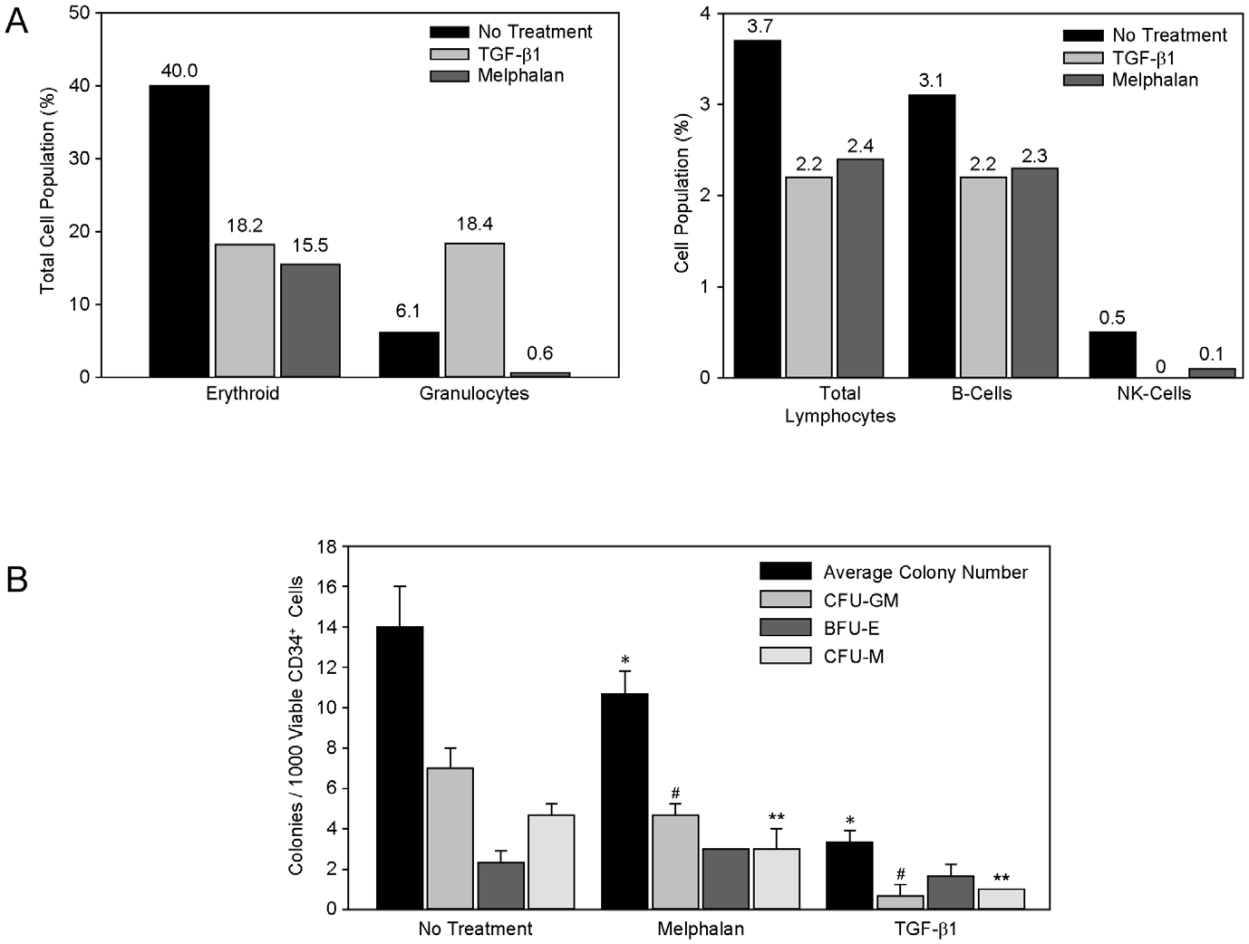
Melphalan or rTGF-β1 exposure diminished the ability of HOB to support CD34+ bone marrow cells. **A**) To evaluate HOB support of CD34+ bone marrow cells, HOB cells were exposed to melphalan [25 µg/ml] or rTGF-β1 [10 ng/ml] for 24 hours. After the 24 hour exposure, the HOB were thoroughly rinsed and CD34+ cells (8.8×10^5^) were added in co-culture. Recombinant IL-3 (100 ng/ml) was added in all groups. CD34+ cells were collected after co-culture for 48 hours and the samples were analyzed for the expression of cell surface markers. **B**) HOB were exposed to melphalan [50 µg/ml] or rTGF-β1 [10 ng/ml] for 24 hours. The HOB layers were rinsed and CD34+ bone marrow cells (1.75×10^5^) were added in co-culture for 48 hours. The CD34+ cells were collected from the HOB layers, viability determined, and 1×10^3^ viable CD34+ bone marrow cells were added to methocult containing factors for myeloid lineage in triplicate. Colonies were counted and scored after 7 days in culture. *p<0.05 (average number of colonies, treated vs untreated), **p<0.05 (CFU-GM colonies, treated vs untreated) and #p<0.05 (CFU-M colonies, treated vs untreated).

One of the critical functions of HOB in the endosteal niche is to support stem cells for appropriate hematopoietic reconstitution to occur post-transplantation [31]. Therefore, we investigated the effects of melphalan, VP-16 and rTGF-β1 on the ability of HOB to support CD34+ hematopoietic stem/progenitor cell self-renewal and lineage differentiation. Figure 2A summarizes observations indicating that following exposure to rTGF-β1 or melphalan, distinct differentiation patterns of CD34+ bone marrow cells were observed. Deficits in total lymphocytes, with specific alteration of B cell and NK cell differentiation were observed following co-culture with damaged HOB. T-cells represented 0.1% of the lymphocyte cell population in the untreated group, while no T cells were detected in cultures that included HOB treated with rTGF-β1 or melphalan (data not shown). Additionally, melphalan exposure of HOB led to a decreased granulocyte population relative to total cell numbers, while exposure to rTGF-β1 enhanced granulocyte differentiation. Both rTGF-β1 and melphalan exposure decreased erythrocyte progenitors. There was no change in monocytes with rTGF-β1 pre-exposure of HOB layers and a slight decrease in monocytes with melphalan pre-exposure (data not shown). Viability among all groups was similar (82–87%) and the total number of CD34+ blasts was comparable between all treatment groups.

To evaluate the ability of damaged HOB to support hematopoietic progenitors, HOB were exposed to melphalan or rTGF-β1, rinsed and CD34+ bone marrow cells were added in co-culture, and colony assays completed. We determined that melphalan and rTGF-β1 pre-exposure of HOB led to significant decreases in colony forming units-granulocyte macrophage (CFU-GM) and colony forming units-monocyte (CFU-M) colonies formed from CD34+ progenitor cells.

### Exposure of HOB to chemotherapeutic agents or rTGF-β1 diminishes HOB support of human embryonic stem cells and adhesion of pro-B cells

**Figure 3:**
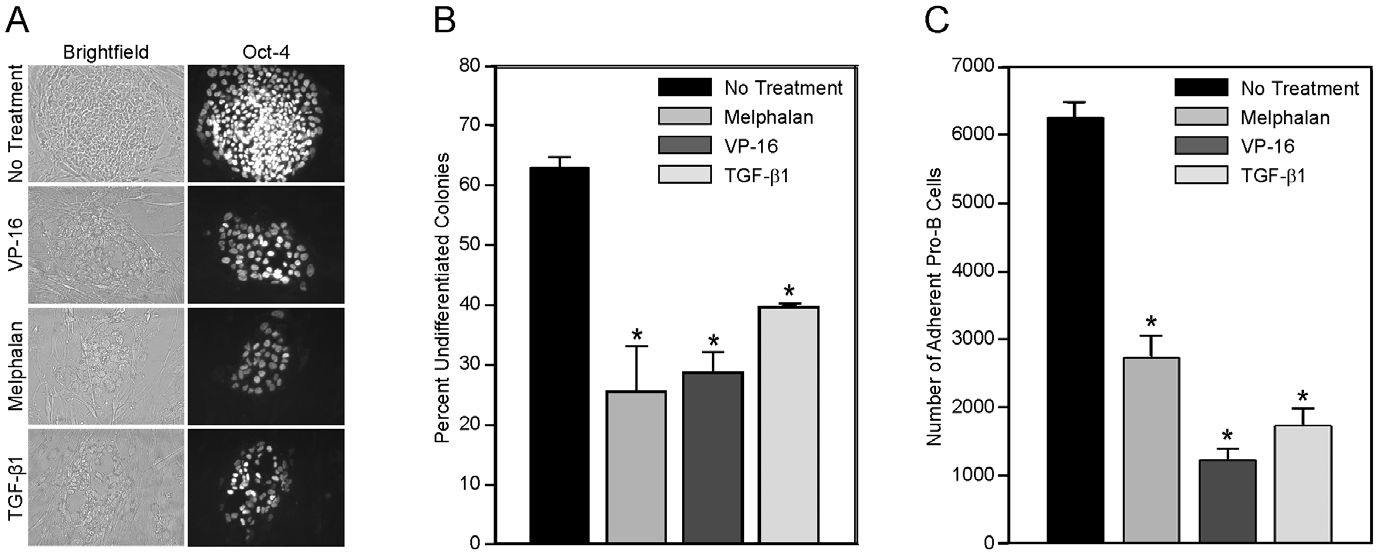
Exposure of HOB to chemotherapeutic agents or rTGF-β1 diminished the ability of HOB to support human embryonic stem cells and decreased pro-B cell interaction with HOB. **A**) HOB were pre-exposed to melphalan [50 µg/ml], VP-16 [50 µM] or rTGF-β1 [10 ng/ml] for 24 hours. The HOB were rinsed thoroughly and human embryonic stem cells were added in co-culture with the HOB. After 2 days of co-culture, stem cell colonies were counted, stained for Oct-4 as an indicator of pluripotent potential and **B**) designated as undifferentiated or differentiated based on classic morphology of well defined borders. **C**) To determine if exposure of HOB to chemotherapeutic agents or rTGF-β1 affected pro-B cell adherence, HOB were exposed to melphalan [50 µg/ml], VP-16 [50 µM] or rTGF-β1 [10 ng/ml] for 24 hours. Adherent layers of HOB were rinsed thoroughly and co-cultured with fluorescently labeled pro-B cells (1×10^5^). After 24 hours the media was aspirated and non-adherent pro-B cells were gently rinsed. Remaining HOB and pro-B cells adherent to the HOB layer were trypsinized and events counted on high flow rate for 30 seconds to enumerate number of pro-B cells attached to the HOB. *p<0.05.

Based on the previous data describing altered support of hematopoietic progenitors, we also investigated how chemotherapeutic agents or rTGF-β1 modulated the ability of HOB to support human embryonic stem cells as an additional read out of altered HOB function. To evaluate markers of pluripotent potential, Oct-4 staining was completed on embryonic stem cell colonies co-cultured with HOB pre-exposed to chemotherapeutic agents or rTGF-β1. Oct-4 staining decreased in those colonies grown on HOB exposed to the different agents (Figure 3A). Data shown in Figure 3B suggests that healthy HOB have the ability to support undifferentiated stem cell colonies characterized by morphology of dense round colonies with definitive, regular cell borders. In contrast, there were increased numbers of differentiated colonies, defined by irregular borders, on coverslips where colonies were grown on HOB layers exposed to melphalan, VP-16 or rTGF-β1.

Alterations in HOB function after aggressive treatment could impact transplant engraftment and hematopoietic reconstitution [8], [24]. We utilized well characterized non-adherent CXCR-4+/VLA-4+ C1.92 and JM-1 pro-B cells as a tool to investigate the effects of chemotherapeutic agents or rTGF-β1 on the ability of HOB to support immature hematopoietic progenitor cell adhesion. Experiments summarized in Figure 3C indicate that following HOB exposure to chemotherapeutic agents or rTGF-β1, C1.92 pro-B cells do not adhere as efficiently as they do to HOB layers not exposed to those agents. To determine if alterations in adhesion molecule expression were associated with decreased adhesion between C1.92 and HOB, VCAM-1, CD44 and hyaluronan expression were evaluated on the HOB in the presence and absence of exposure to chemotherapeutic agents or rTGF-β1. No modulation of these proteins was detected on the HOB during exposure to these agents (data not shown).

### HOB expression of CXCL-12 is diminished following exposure to chemotherapeutic agents or rTGF-β1

**Figure 4:**
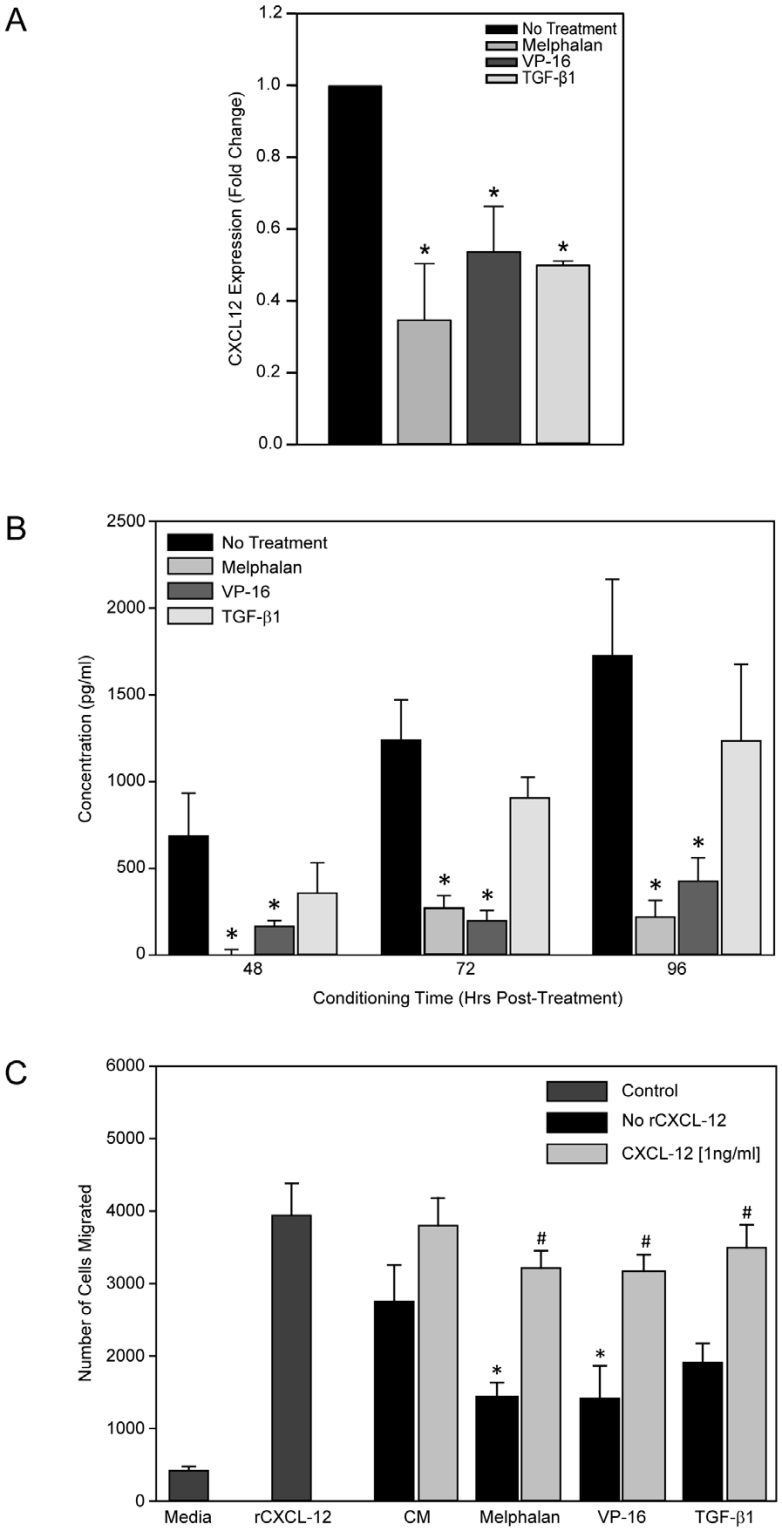
Exposure of HOB to chemotherapeutic agents or rTGF-β1 decreased the expression of CXCL-12 and diminished support of pro-B cell chemotaxis. **A**) HOB were exposed to melphalan [50 µg/ml], VP-16 [50 µM] or rTGF-β1 [10 ng/ml] for 24 hours. RNA was isolated and real time RT-PCR completed for CXCL-12. **B**) HOB were exposed to melphalan [50 µg/ml], VP-16 [50 µM] or rTGF-β1 [10 ng/ml] for 24 hours. Following exposure, cells were rinsed and new media added and conditioned for 48, 72 or 96 hours. Supernatants were collected and evaluated by CXCL-12 ELISA. **C**) HOB were exposed to melphalan [50 µg/ml], VP-16 [50 µM] or rTGF-β1 [10 ng/ml] for 24 hours. Following exposure, 350 ul of supernatant was placed into the bottom chamber of a transwell with or without CXCL-12 [1 ng/ml]. HOB media and CXCL-12 [100 ng/ml] were used as negative and positive controls, respectively. JM-1 progenitor cells (1.5×10^5^) were placed in the top chamber and allowed to migrate for 4 hours. After 4 hours, the cells migrating to the bottom chamber were read on high flow rate for 30 seconds on a FACSCalibur flow cytometer. (CM = conditioned media), *p<0.05 (CM compared to treatments), #p<0.05 (individual group compared to CXCL-12 addition).

Inhibition of CXCL-12 in the bone marrow has been shown to have a negative impact on chemotaxis coincident with deficits in HSC homing and engraftment [18], [32]. To further investigate the impact of melphalan, VP-16 or rTGF-β1 exposure on expression of HOB derived CXCL-12, real time RT-PCR and ELISA were completed as described. Exposure of HOB to chemotherapeutic agents or rTGF-β1 decreased the amount of CXCL-12 mRNA and protein detected by real time RT-PCR and ELISA, respectively (Figure 4A and B). Jung et al. have described the regulation of CXCL-12 expression in HOB and the effect on homing and reported that treatment of HOB with TGF-β decreased CXCL-12 secretion, consistent with our results [33]. Additionally, as a functional readout of a potential CXCL-12 deficit in our model, chemotaxis assays were completed. Figure 4C summarizes data indicating that the chemotaxis of JM-1 cells toward adherent layers of HOB was impaired following pre-exposure of HOB to melphalan, VP-16 and rTGF-β1. The addition of CXCL-12 [1 ng/ml] to the media of HOB pre-exposed to chemotherapeutic agents or rTGF-β1 prior to the start of the assay restored the chemotaxis of JM-1 cells. As an additional control, AMD-3100, a CXCR-4 inhibitor, was used to confirm that pre-incubation of JM-1 cells with AMD-3100 prior to the start of chemotaxis inhibited migration towards both rCXCL-12 as well as media conditioned by HOB (data not shown).

### Direct and indirect exposure to chemotherapeutic agents or rTGF-β1 results in global changes in HOB gene expression

**Figure 5:**
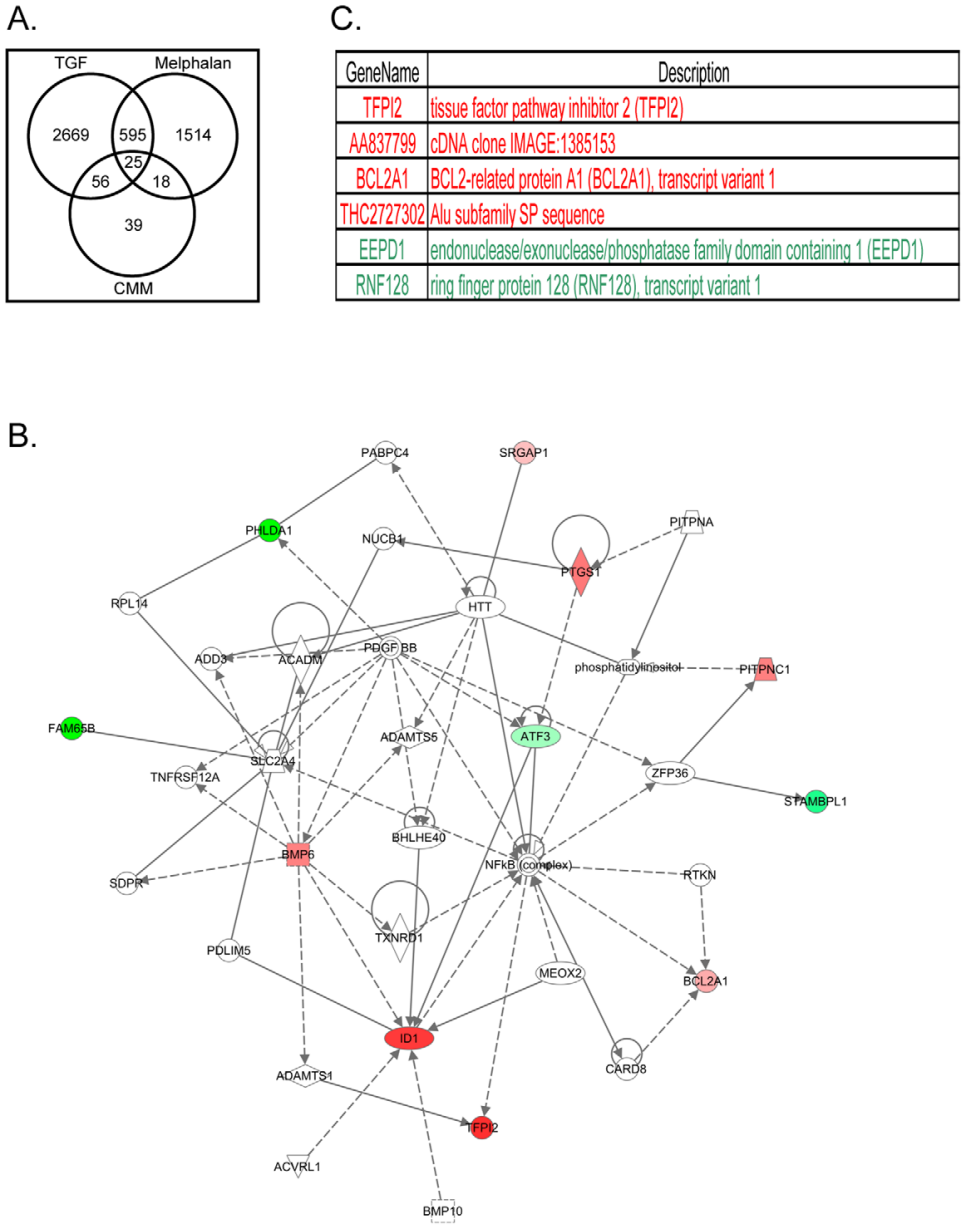
Global changes in gene expression in HOB following direct and indirect exposure to chemotherapeutic agents or rTGF-β1. HOB cells were exposed to melphalan [50 µg/ml], rTGF-β1 [10 ng/ml] or conditioned media from BMSC (CMM) pre-exposed to melphalan [50 µg/ml-24 hours] for 6 hours. BMSC exposed to melphalan were rinsed and fresh media was place on adherent layers to condition prior to stimulating HOB. After the 6 hour exposure, HOB RNA was isolated and microarray analysis completed to evaluate global changes in gene expression. **A**) The Venn diagram summarizes the number of HOB genes that changed within each group as well as the changes between the groups. **B**) A network diagram was generated for the intersection of all 3 treatment groups highlighting the convergence of potential pathways associated with the genes found in common between the 3 treatment groups, such as NF-κB. All genes listed were generated using a 2.5% FDR and >1.5 fold change in significance. **C**) A gene summary was made common genes up-regulated (4, red) or down-regulated (2, green) for the intersection of all 3 treatments (rTGF-β1: CMM: melphalan).

To elucidate the global changes that occur in HOB with direct and indirect insult from chemotherapeutic agents or rTGF-β1, HOB were exposed to melphalan or rTGF-β1. In addition, HOB were exposed to conditioned media from BMSC that had been pre-exposed to melphalan (drug removed prior to collection of conditioned media) to recapitulate signaling that may occur in response not only to active TGF-β1 elaborated by BMSC, but also in response to the collective soluble factors elaborated by BMSC in response to stress induced by chemotherapeutic agents. Microarray analysis of gene expression was performed as described. The genes for which expression changed in each group individually, and common gene targets that overlap between treatment groups, are indicated in the Venn diagram (Figure 5A). HOB exposure to rTGF-β1 resulted in the highest number of genes influenced across the groups evaluated, with melphalan exposure also resulting in a robust effect. Twenty-five common genes significantly changed when the intersection of all treatment groups was considered. The Venn and network diagrams show the number of genes modulated in response to exposure to melphalan or rTGF-β1, and potential relationships between some of the responsive genes, as well as convergence on signaling molecules such as the NF-κB complex which emerged as a hub of signaling (Figure 5B). The genes that were commonly up-regulated (red) or down-regulated (green) between treatment groups are shown and include: 4 up-regulated, 2 down-regulated (Figure 5C, intersection of all 3 treatments), 16 up-regulated, 3 down-regulated (Figure S1, CMM: melphalan), 26 up-regulated, 11 down-regulated (Figure S2, rTGF:CMM), and 97 up-regulated, 188 down-regulated (Figure S3, rTGF: melphalan). This summary can be compared with those genes that were influenced, but in opposing directions, between groups (Table S1).

## Discussion

Myelosuppressive and ablative therapies followed by stem cell transplantation is used to treat hematopoietic, breast, ovarian and brain tumors as well as childhood sarcomas, and immune deficiencies [34], [35]. As the primary site of postnatal hematopoiesis, the functional integrity of the bone marrow microenvironment is critical for hematopoietic recovery. Earlier reports have suggested that BMSC are vulnerable to functional damage imposed by aggressive chemotherapeutic agents [16], [17], [36]. These studies have focused largely on the ability of stromal cells to generate fibroblastic colonies (CFU-F) or to support survival or expansion of committed progenitor cells when isolated from patients following drug exposures [37].

Murine models of ablative treatment and stem cell transplantation have shown long-term deficits in hematopoietic recovery and *in vitro* models have paralleled these studies documenting the inability of transplanted cells to migrate efficiently to the anatomical niches necessary for engraftment [38]. Observations of long-term hematopoietic deficits in the bone marrow of transplantation patients suggest that the functionality of the developmental niches required for appropriate support of immature hematopoietic or stem cells may have be compromised by aggressive pre-transplant therapies. One study observed that at 1 year post transplant 61% of patients had subnormal values in one or more hematopoietic lineages [39]. Furthermore, Nieboer et al. showed that at 5 years post transplant 15% of the patient population analyzed had low values in one or more hematopoietic cell lineages [40]. Investigations of the mechanisms that underlie damage of the hematopoietic and stem cell niches are further encouraged by retrospective studies of patients that received allogeneic bone marrow transplants in which patient HSC numbers did not recover to the levels of control patients, even after 12 years, as measured by CFU-F frequencies, suggesting that the damage of the structural, hematopoietic supportive cells of the bone marrow can be sustained [41].

In the current study we characterized the impact of direct and indirect damage on HOB and their subsequent ability to support progenitor and stem cells. Following transplantation and during development, HSC home to the endosteal niche which acts as a critical regulator of stem cell quiescence, proliferation, and conservation of the stem cell pool. This stem cell pool also provides all the cells necessary for hematopoietic reconstitution and recovery. Direct contact between HOB and HSC is required for HSC survival [7], [8], [42] with a dynamic relationship demonstrated by the ability of HSC to regulate the cytokines expressed by HOB in order to enhance their own survival. Studies by Calvi et al., and others, have shown that number the of osteoblasts present in the niche directly modulates the numbers of HSC supported by the niche [7], [8], [24].

Our data shows that direct exposure to chemotherapeutic agents increases the activity of HOB derived TGF-β1, one of the known negative regulators of HSCs (Figure 1) [15]–[17]. Consistent with the literature suggesting that TGF-β activity leads to decreased expression of HSC surface cytokine receptors and a deficit in the stem cell pool [17], [43], Figure 2 summarizes data showing HOB pre-exposed to either melphalan or rTGF-β1 had a decreased ability to support appropriate differentiation of CD34+ bone marrow cells. Alterations occurred in the lymphocyte population, with decreased B cells, NK cells and T cells. In addition there were decreased erythroid and granulocyte progenitors derived from CD34+ cells co-cultured with pre-exposed HOB. The total CD34+ blast population was similar between all three treatment groups, suggesting damage induced by chemotherapeutic agents may predominately alter HOB modulation of hematopoietic cell differentiation. We also observed that CD34+ bone marrow cells co-cultured with HOB exposed to melphalan or rTGF-β1 had decreased hematopoietic potential when evaluated by a colony assay with an overall decrease in colony number, specifically CFU-GM and CFU-M colonies. Interestingly, it was observed that BFU-E colony formation was not significantly altered, while surface staining showed decreased erythroid progenitors. This suggests that the more differentiated erythroid cells may be affected by damaged microenvironment components, while the more primitive erythroid precursors are spared. Figure 3 provides additional evidence that damaged HOB have a decreased ability to interact with, and support, both human embryonic stem cells as well as more differentiated pro-B cells. Direct treatment of HOB with chemotherapeutic agents or rTGF-β1 resulted in a reduction of pro-B cell adhesion as well as a diminished ability to support Oct-4 positive embryonic stem cells.

An important consideration when evaluating the dynamic balance of the niche is the role of adhesion molecules physically tethering progenitor cells to supportive cells of the bone marrow, providing signals for their maturation and survival. Earlier reports have described the role of the VCAM-1/VLA-4 interaction in hematopoiesis. Ryan et al. demonstrated that adhesion of B-cell precursors to BMSC was dependent on this interaction and Dittel et al. elucidated how cytokines could alter the surface expression of VCAM-1 [44], [45].

In addition to the VCAM-1/VLA-4 interaction, the CD44/hyaluronan (HA) interaction has also been recognized for its role in hematopoiesis and homing of primitive cells to the bone marrow [46]. Avigdor et al. demonstrated the important roles of CXCL-12 with respect to migration and anchoring of progenitors to the bone marrow through CD44/HA [47].. It was based on these observations that we investigated the effects of chemotherapeutic agents on VCAM-1, CD44, and HA in our model of HOB damage. Figure 3C shows that pro-B cells co-cultured with HOB pre-exposed to melphalan, VP-16 or rTGF-β1 are unable to adhere to the HOB efficiently. However, investigation of the adhesion molecules VCAM-1, CD44 and HA indicated no altered expression following exposure to chemotherapeutic agents or rTGF-β1 (data not shown). These observations suggest that the deficit in hematopoietic support, in our model, may be the result of changes in a soluble factor acting in either an autocrine or paracrine manner to diminish optimal cell-cell interactions or that an unidentified adhesion molecule is influenced by the chemotherapeutic agents evaluated. Paracrine effects could be modulated, in part, through alteration of integrin activity, which would not have been detected in our assay. These observations also suggest very specific effects of chemotherapeutic agents on BMSC and HOB components of the niche, emphasizing the need to understand each population individually to understand the total response of the niche to therapy.

We next examined the effect of chemotherapeutic agents or rTGF-β1 on HOB derived CXCL-12. Data in Figure 4A and B shows that melphalan, VP-16 and rTGF-β1 decreased CXCL-12 mRNA and protein expression in HOB. Jung et al. have previously shown that CXCL-12 secreted by HOB may contribute to stem cell homing [48]. Decreased CXCL-12 in the microenvironment due to chemotherapy damage of HOB may affect hematopoietic reconstitution. The decrease in CXCL-12 protein expression correlated with decreased chemotaxis of CXCR-4+ JM-1 cells towards HOB pre-exposed to melphalan, VP-16 or rTGF-β1. The decrease in chemotaxis was restored with the addition of CXCL-12, provide further evidence that damage to HOB may affect stem cell homing. CXCL-12 is not the only factor critical for homing and further investigation of other factors, including stem cell factor (SCF), which has been shown to synergize with CXCL-12 in homing of stem cells and retention in their developmental niche need to be investigated [49].

Collectively, these data suggest that generation of active TGF-β1 in the endosteal niche can negatively affect production of CXCL-12, thus impairing progenitor cell homing to the bone marrow, and subsequent engraftment, and reconstitution of the patient's immune system. This observation of altered gene expression in HOB resulting from exposure to chemotherapeutic agents prompted us to use microarray analysis to investigate the magnitude of direct and indirect damage to HOB induced by exposure to melphalan or rTGF-β1. After 6 hours of exposure to those agents, the diverse changes observed in HOB gene expression alone allow for a better understanding of the significance of the potential damage to the niche and the subsequent impact on hematopoietic reconstitution that relies on balanced expression of several proteins. Interestingly, exposure of HOB to rTGF-β1, melphalan and conditioned media from melphalan pre-exposed BMSC all identified NF-κB as a point of convergence (Figure 5B). As with all gene expression pathway analysis, in the absence of targeted genetic manipulation or biochemical analysis, the interactions remain hypothetical. The most pronounced value of these data in the current study is to provide a sense of the responsiveness of HOB to genotoxic stress, as well as to factors such as TGF-β1, which may participate in both autocrine and paracrine signaling in the bone marrow niche. Points of convergence, such as NF-κB, may then provide the focus for a more mechanistic investigation of cell signaling downstream of stress in the bone marrow microenvironment. Of relevance to the current investigation, subsequent real-time PCR analyzing RUNX2, alkaline phosphatase, collagen 1A1 and osteocalcin demonstrated distinct responses to melphalan and VP-16 exposure. The most consistent changes observed in pilot studies were decreases in RUNX2 and collagen 1A1, with the most pronounced reduction for genes observed following melphalan exposure (data not shown). No consistent change in osteocalcin or alkaline phosphatase was noted. The potential influence of dose escalated chemotherapy on osteoblast differentiation is currently under further investigation by our group.

Central to our investigation was an interest in the influence of active TGF-β1 released from HOB exposed to chemotherapeutic agents as well as TGF-β1 that may be released from neighboring BMSC in a damaged microenvironment as just two potential sources of this growth factor. Studies by Batard et al. have described the importance of low levels of TGF-β1 in the bone marrow microenvironment for maintenance of the stem cell pool through up-regulation of the CD34 antigen, a marker of primitive HSC [43]. Consistent with the need for rigorous control of total TGF-β1 levels, a number of studies have shown the benefit of TGF-β neutralization during therapy [50]. Lagneaux et al. showed that stromal cells isolated from B-CLL patients had increased TGF-β production correlating with decreased colony-stimulating activity which was corrected by neutralizing TGF-β activity [17]. Using a murine model of breast cancer, Biswas et al. showed that radiation or doxorubicin treatment increased levels of TGF-β which correlated with increased circulating tumor cells and increased metastasis [51]. These effects were abrogated by anti-TGF-β antibodies, providing rationale for utilization of TGF-β inhibitors, such as GC1008, which is currently in clinical trials in the setting of renal cell carcinoma and malignant melanoma [52]. Based on our observations, application of TGF-β neutralizing antibodies may have utility through influence on both hematopoietic cells and the niche in which they develop.

Our observations indicate that HOB are susceptible to genotoxic stress documented by alteration of gene expression profiles (Figure 5, Figure S1, S2, S3, Table S1) and functional deficits in hematopoietic cell support, including differentiation and hematopoietic potential (Figure 2). Further investigation will identify targets that may prove useful in augmenting hematopoietic recovery through “balancing” the stem cell niche following therapy-induced damage. Long-term hematopoietic deficits may be derived from the immediate changes in the niche that are imposed by aggressive therapeutic regimens. This aspect of altered bone marrow function following exposure to chemotherapeutic agents may highlight an area in which a better understanding of HOB damage by genotoxic agents may identify new therapeutic strategies to augment efficient patient recovery following bone marrow transplantation.

## Supporting Information

Figure S1 **Soluble factors in BMSC exposed to chemotherapy induce HOB gene expression changes in common with those subsequent to melphalan exposure.** HOB cells were treated for 6 hours with 50 µg/ml melphalan, or conditioned media from BMSC pre-treated with 50 µg/ml melphalan for 24 hours. BMSC exposed to melphalan were rinsed and fresh media was place on adherent layers to condition and to remove drug prior to stimulating HOB. After the 6 hour treatment, HOB RNA was isolated and microarray analysis was completed to evaluate global changes in gene expression. **A**) Gene changes for the intersections of the CMM:melphalan groups were analyzed based on the genes that commonly increased (16, red) or decreased (3, green). **B**) A network diagram was generated for the intersection of CMM:melphalan groups that highlights the convergence of potential pathways between these 2 treatment groups. All genes listed were generated using a 2.5% FDR and 1.5 fold significant cut off.
(PDF)

Figure S2 **Soluble factors in BMSC exposed to chemotherapy induce HOB gene expression changes in common with those subsequent to rTGF-β exposure.** HOB cells were treated for 6 hours with 10 ng/ml rTGF-β, or conditioned media from BMSC pre-treated with 50 µg/ml melphalan for 24 hours. BMSC exposed to melphalan were rinsed and fresh media was place on adherent layers to condition and to remove drug prior to stimulating HOB. After the 6 hour treatment, HOB RNA was isolated and microarray analysis was completed to evaluate global changes in gene expression. **A**) Gene changes for the intersections of the TGF-β:CMM groups were analyzed based on the genes that commonly increased (26, red) or decreased (11, green). **B**) A network diagram was generated for the intersection of TGF-β:CMM groups that highlights the convergence of potential pathways between these 2 treatment groups. All genes listed were generated using a 2.5% FDR and 1.5 fold significant cut off.
(PDF)

Figure S3 **rTGF-β exposure induces HOB gene expression changes in common with those subsequent to melphalan exposure.** HOB cells were treated for 6 hours with 10 ng/ml rTGF-β, or with 50 µg/ml melphalan for 24 hours. After the 6 hour treatment, HOB RNA was isolated and microarray analysis was completed to evaluate global changes in gene expression. **A**) Gene changes for the intersections of the TGF-β:melphalan groups were analyzed based on the genes that commonly increased (97, red) or decreased (188, green). **B**) A network diagram was generated for the intersection of TGF-β: melphalan groups that highlights the convergence of potential pathways between these 2 treatment groups. All genes listed were generated using a 2.5% FDR and 1.5 fold significant cut off.
(PDF)

Table S1 **Global changes occur in gene expression with direct and indirect chemotherapy treatment.** Complete lists of genes modified, in the same or opposite directions, in response to treatment for each intersection group are shown (CMM:melphalan, CMM:TGF, TGF:melphalan, Intersection of all 3) with fold changes and confidence intervals (C.I.). All genes listed were generated using a 2.5% FDR and 1.5 fold significant cut off.
(XLS)
