# Supplementary material for: Bone Marrow Osteoblast Damage by Chemotherapeutic Agents
Source: PLoS One. 2012 Feb 17;7(2):e30758. doi: 10.1371/journal.pone.0030758 (PMC3281873; doi:10.1371/journal.pone.0030758)
Supplement: Figure S1 — Soluble factors in BMSC exposed to chemotherapy induce HOB gene expression changes in common with those subsequent to melphalan exposure. HOB cells were treated for 6 hours with 50 µg/ml melphalan, or conditioned media from BMSC pre-treated with 50 µg/ml melphalan for 24 hours. BMSC exposed to melphalan were rinsed and fresh media was place on adherent layers to condition and to remove drug prior to stimulating HOB. After the 6 hour treatment, HOB RNA was isolated and microarray analysis was completed to evaluate global changes in gene expression. A) Gene changes for the intersections of the CMM:melphalan groups were analyzed based on the genes that commonly increased (16, red) or decreased (3, green). B) A network diagram was generated for the intersection of CMM:melphalan groups that highlights the convergence of potential pathways between these 2 treatment groups. All genes listed were generated using a 2.5% FDR and 1.5 fold significant cut off. (PDF) [file pone.0030758.s001.pdf]

A.

GeneName	Description
THC2673554	Alu subfamily J sequence
AF227517	sprouty-4C mRNA, complete cds. [AF227517]
IL1B	interleukin 1, beta (IL1B), mRNA [NM_000576]
BCL2A1	BCL2-related protein A1 (BCL2A1), transcript variant 1, mRNA [NM_004049]
A_32_P206561	Unknown
CCL20	chemokine (C-C motif) ligand 20 (CCL20), mRNA [NM_004591]
NR4A3	nuclear receptor subfamily 4, group A, member 3 (NR4A3), transcript variant 2, mRNA [NM_173198]
RASD1	RAS, dexamethasone-induced 1 (RASD1), mRNA [NM_016084]
THC2674068	Q4S8B2_TETNG (Q4S8B2) Chromosome undetermined SCAF14706
AA837799	cDNA clone IMAGE:1385153, mRNA sequence [AA837799]
THC2560068	High mobility group protein HMGI-C (High mobility group AT-hook protein 2)
THC2727302	Alu subfamily SP sequence
LOC390595	cDNA FLJ13740 fis, clone PLACE3000199. [AK023802]
PHLDA1	pleckstrin homology-like domain, family A, member 1 (PHLDA1), mRNA [NM_007350]
ANKRD10	ankyrin repeat domain 10 (ANKRD10), mRNA [NM_017664]
AY831680	HC1 (HC1) mRNA, complete cds. [AY831680]
NUAK1	NUAK family, SNF1-like kinase, 1 (NUAK1), mRNA [NM_014840]
EEPD1	endonuclease/exonuclease/phosphatase family domain containing 1 (EEPD1)
RNF128	ring finger protein 128 (RNF128), transcript variant 1

B.

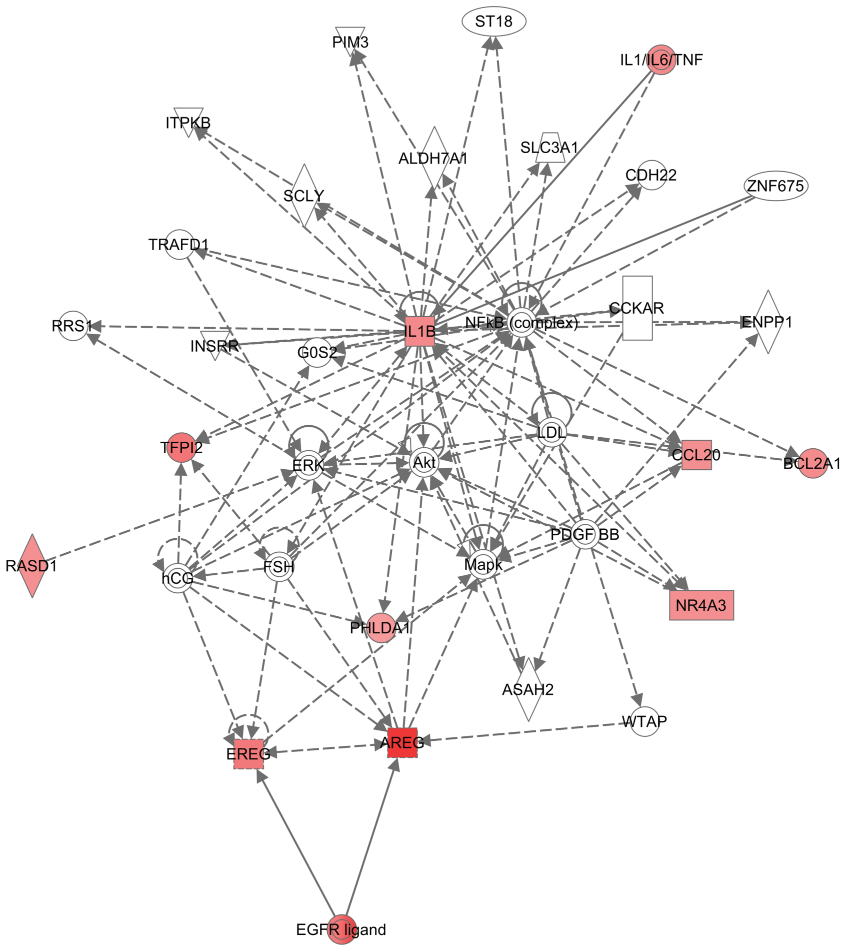

Supplemental figure 1
