# Supplementary material for: Bone Marrow Osteoblast Damage by Chemotherapeutic Agents
Source: PLoS One. 2012 Feb 17;7(2):e30758. doi: 10.1371/journal.pone.0030758 (PMC3281873; doi:10.1371/journal.pone.0030758)
Supplement: Figure S2 — Soluble factors in BMSC exposed to chemotherapy induce HOB gene expression changes in common with those subsequent to rTGF-β exposure. HOB cells were treated for 6 hours with 10 ng/ml rTGF-β, or conditioned media from BMSC pre-treated with 50 µg/ml melphalan for 24 hours. BMSC exposed to melphalan were rinsed and fresh media was place on adherent layers to condition and to remove drug prior to stimulating HOB. After the 6 hour treatment, HOB RNA was isolated and microarray analysis was completed to evaluate global changes in gene expression. A) Gene changes for the intersections of the TGF-β:CMM groups were analyzed based on the genes that commonly increased (26, red) or decreased (11, green). B) A network diagram was generated for the intersection of TGF-β:CMM groups that highlights the convergence of potential pathways between these 2 treatment groups. All genes listed were generated using a 2.5% FDR and 1.5 fold significant cut off. (PDF) [file pone.0030758.s002.pdf]

A.

GeneName	Description
TFPI2	tissue factor pathway inhibitor 2 (TFPI2)
AA837799	cDNA clone IMAGE:1385153, mRNA sequence [AA837799]
ID1	inhibitor of DNA binding 1, dominant negative helix-loop-helix protein (ID1), transcript variant 1
PTGS1	prostaglandin-endoperoxide synthase 1 (prostaglandin G/H synthase and cyclooxygenase) (PTGS1), transcript variant 1
PITPNC1	phosphatidylinositol transfer protein, cytoplasmic 1 (PITPNC1), transcript variant 2
BMP6	bone morphogenetic protein 6 (BMP6)
CCL7	chemokine (C-C motif) ligand 7 (CCL7)
GEM	GTP binding protein overexpressed in skeletal muscle (GEM), transcript variant 1
FLJ14213	protor-2 (FLJ14213)
DIRAS3	DIRAS family, GTP-binding RAS-like 3 (DIRAS3)
MMP13	matrix metallopeptidase 13 (collagenase 3) (MMP13)
HAS1	hyaluronan synthase 1 (HAS1)
BCL2A1	BCL2-related protein A1 (BCL2A1), transcript variant 1
BDKRB1	bradykinin receptor B1 (BDKRB1)
DB381305	cDNA clone PLACE3000400 3', mRNA sequence [DB381305]
MYB	v-myb myeloblastosis viral oncogene homolog (avian) (MYB)
IFNE1	interferon epsilon 1 (IFNE1)
FGF16	fibroblast growth factor 16 (FGF16)
SRGAP1	SLIT-ROBO Rho GTPase activating protein 1
THC2727302	Alu subfamily SP sequence
AL390214	cDNA DKFZp564O2423 (from clone DKFZp564O2423) [AL390214]
PRL	prolactin (PRL)
SPON1	spondin 1, extracellular matrix protein (SPON1)
GPRC5A	G protein-coupled receptor, family C, group 5, member A (GPRC5A)
AA293788	cDNA clone IMAGE:727217 5' similar to gb:X02761_cds1 FIBRONECTIN PRECURSOR (HUMAN)
AF086511	cDNA clone ZE03A08. [AF086511]
GREM1	gremlin 1, cysteine knot superfamily, homolog (Xenopus laevis) (GREM1)
ATF3	activating transcription factor 3 (ATF3), transcript variant 4
L3MBTL2	cDNA FLJ32615 fis, clone STOMA2000148. [AK057177]
C1QTNF7	C1q and tumor necrosis factor related protein 7 (C1QTNF7)
HSPB3	heat shock 27kDa protein 3 (HSPB3)
PRKAG3	protein kinase, AMP-activated, gamma 3 non-catalytic subunit (PRKAG3)
EEPD1	endonuclease/exonuclease/phosphatase family domain containing 1 (EEPD1)
RNF128	ring finger protein 128 (RNF128), transcript variant 1
KIAA1199	KIAA1199 (KIAA1199)
LOC283143	cDNA FLJ33283 fis, clone ASTRO2009177. [AK090602]
KLF5	Kruppel-like factor 5 (intestinal) (KLF5)

B.

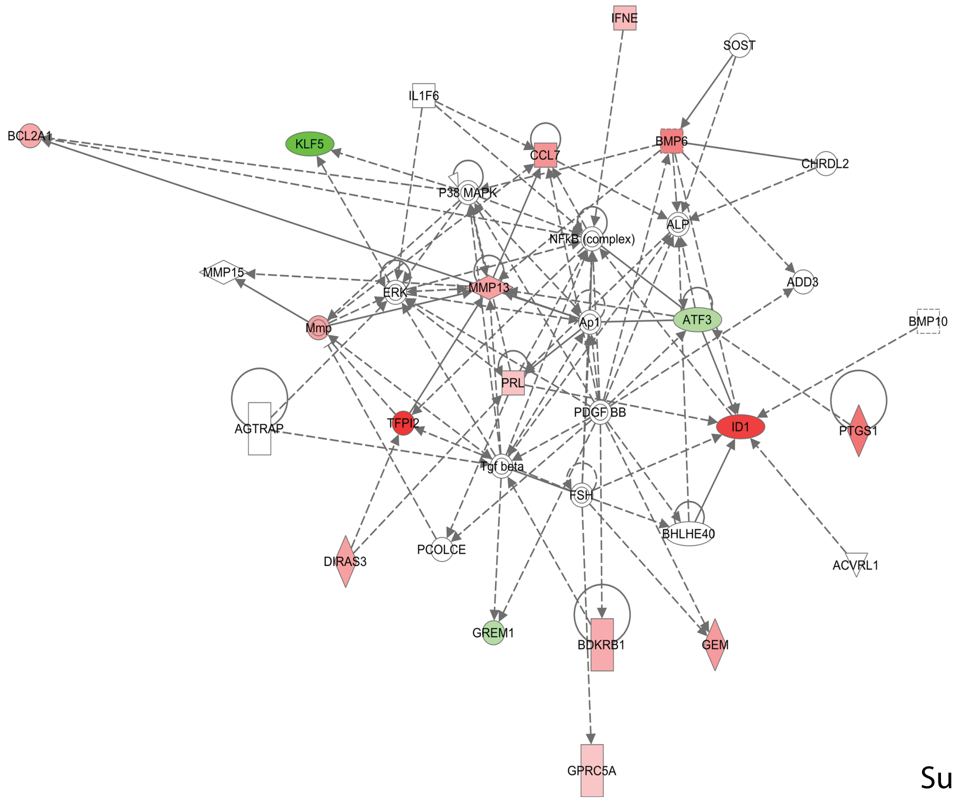

Supplemental figure 2
