# Supplementary material for: Bone Marrow Osteoblast Damage by Chemotherapeutic Agents
Source: PLoS One. 2012 Feb 17;7(2):e30758. doi: 10.1371/journal.pone.0030758 (PMC3281873; doi:10.1371/journal.pone.0030758)
Supplement: Figure S3 — rTGF-β exposure induces HOB gene expression changes in common with those subsequent to melphalan exposure. HOB cells were treated for 6 hours with 10 ng/ml rTGF-β, or with 50 µg/ml melphalan for 24 hours. After the 6 hour treatment, HOB RNA was isolated and microarray analysis was completed to evaluate global changes in gene expression. A) Gene changes for the intersections of the TGF-β:melphalan groups were analyzed based on the genes that commonly increased (97, red) or decreased (188, green). B) A network diagram was generated for the intersection of TGF-β: melphalan groups that highlights the convergence of potential pathways between these 2 treatment groups. All genes listed were generated using a 2.5% FDR and 1.5 fold significant cut off. (PDF) [file pone.0030758.s003.pdf]

A.

GeneName	GeneName	GeneName	GeneName	GeneName	GeneName
PTGS2	C13orf27	AK095141	CF552909	PLEKHA5	IL16
RASL11B	MEX3A	C1orf203	RGMB	BICC1	PHF15
SLC19A2	LOC100129406	MKL2	PHF21A	RAD54B	ARHGAP24
TRIB1	BC063641	LOC653391	BU607658	ANKRD28	GCNT1
LOH3CR2A	DBF4	PRUNE2	ZNF277	SPATA7	JAK2
MEX3B	RGS16	SRGAP2	BC034285	LRIG3	FGF5
CXCR7	KLHDC5	ANKAR	ARHGAP18	LOC100128239	RNF128
TFPI2	CV570707	C6orf165	PXK	DLG3	RUNX1T1
AA837799	CUL3	IKBKE	CXorf57	A_32_P134679	GLI3
FOSB	AMMECR1L	ENST00000311275	TBL1XR1	TACC2	USP53
SNF1LK	BU561469	C6orf167	CC2D2A	IRF2	PSD3
RASL10A	NOL5A	DENND4C	SNX29	TIGD2	CREB5
IER3	C7orf40	RSPO2	FAM108C1	SCN8A	CABLES1
GRIN2C	LOC338756	NFIA	TLR4	FRY	CCDC110
TMEM88	THC2693729	ENST00000219090	PHF3	TBC1D12	EBF1
YRDC	RG9MTD1	MYO1B	IKZF2	ENST00000395742	JAKMIP3
RBM24	BTG2	REV3L	SOCS5	BC030115	PIK3R1
IL12A	LOC285535	KIAA1107	FAT4	AR	FRMD4B
RRAD	EAF1	BC047111	TNRC6B	NRP1	TRERF1
SGK1	THC2713715	PPM1H	OSBPL10	SLC14A1	STAMBPL1
A_32_P219704	B3GNT5	EMX2	C1orf190	ZFHX4	THC2663329
B3GNT2	BC032716	FAM90A1	TET1	PPP2R5A	CIITA
RPL27A	SNORA70	SPATA17	ZNF214	ANKRD34A	SMAD3
BCL2A1	A_32_P109645	C9orf93	TMEM133	KBTBD3	EBF3
CITED4	THC2525241	THSD4	FIGN	LOC157562	TMEM26
HOXD1	BM475547	FUT10	ATP2B1	FAM49A	BNC2
DA116424	CCNJ	EXOC6	ZBTB20	TncRNA	AI827481
SERPINB2	SNORD22	CPNE8	CCDC98	CDC42EP3	IL7
C1orf107	PPIF	TMEM200A	MGC24103	YPEL2	FGD4
THC2619021	LOC85391	STAC	FRMD4A	FOXP2	HGF
MIRHG1	A_32_P105940	BE893137	SHANK2	RGL1	RNF144B
RGS2	C10orf2	PDLIM5	SGMS1	PTPRG	PLEKHA2
BU903025	DUSP2	THC2781306	FLJ33996	KLF12	NHS
USP36	NKRF	DST	HECW2	AK022044	AHRR
NEBL	THC2537217	SPRED1	ENOX1	FGD6	NRG2
MFSD2	SPTY2D1	THC2664860	THC2514262	ARHGAP12	GAS1
CCDC64B	ABT1	ZNF438	KIAA0802	SNTB1	FAM110B
BX375060	THC2729109	KIAA0922	CDC14B	FAM59A	RARB
THC2727164	BC035666	ZNF521	PIK3R3	FMN2	AL080082
THC2669419	T05215	TTC30B	TCF7L1	PHLDB2	ARHGAP20
MESDC1	AF339771	LYST	MLLT3	ACVR1C	TP63
YOD1	CYP27B1	ZSWIM5	ZNF618	ROR1	TNFRSF19
ARC	PLK3	GRK5	LOC284998	AK025909	SEMA6D
ATG16L1	FABP5	HMGA2	MTA3	KALRN	TOX
ZC3H12A	THC2689802	PAG1	PPP2R2B	MITF	KITLG
THC2727302	ARID5A	CNKSR3	EPHA5	PLCL2	
TUFT1	BF575152	MEOX2	MBOAT1	EEPD1	
BM726940	AK022150	BX119882	C6orf32	SHROOM3	

B.

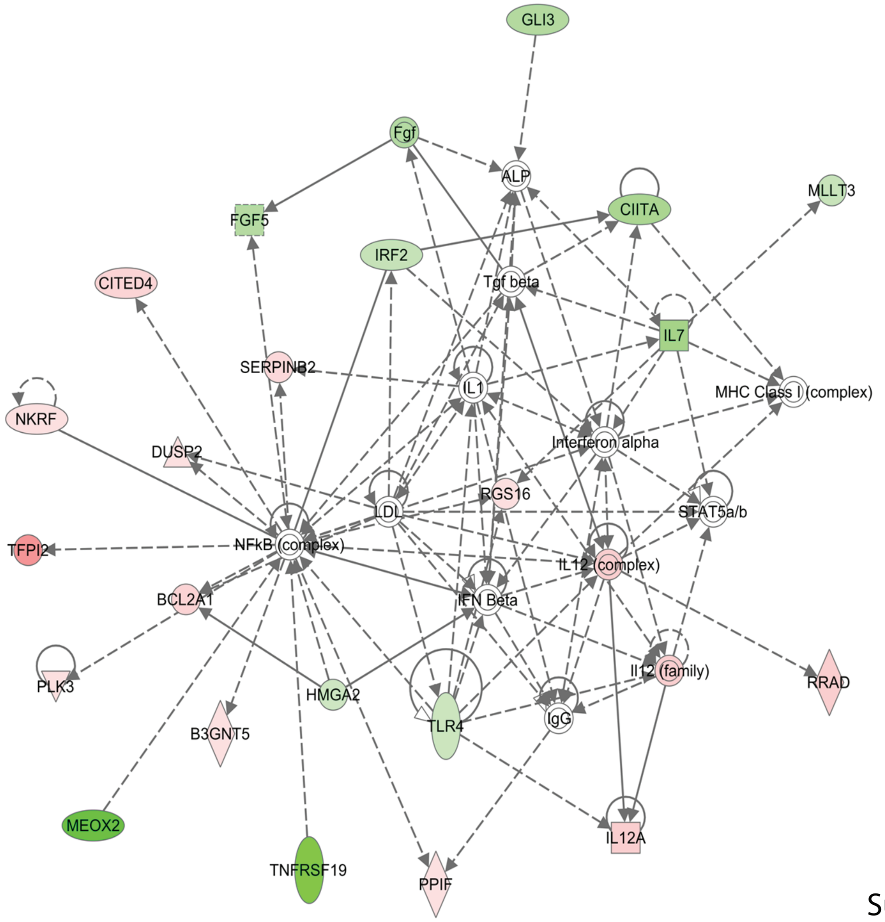

Supplemental figure 3
